# Electroencephalographic and Neuroimaging Asymmetry Correlation in Patients with Attention-Deficit Hyperactivity Disorder

**DOI:** 10.1155/2020/4838291

**Published:** 2020-09-01

**Authors:** M. Longarzo, C. Cavaliere, V. Alfano, G. Mele, M. Salvatore, M. Aiello

**Affiliations:** IRCCS SDN, Via Gianturco 113, 80142 Naples, Italy

## Abstract

The present study explores the correlation between electroencephalographic and neuroimaging asymmetry index from EEG-MRI functional connectome and EEG power analysis in inattention, motion, and mixed profile subgroups of ADHD. Sixty-two subjects from Healthy Brain Network Biobank of the Child Mind Institute dataset were selected basing on the quotient score. From both MRI and EEG asymmetry index, Pearson's correlation, ANOVA, and partial least square analysis were performed matching left and right brain parcels and channels. The asymmetry index significantly correlated across subjects between fMRI and power-EEG in the inattention group in frontal and temporal areas for theta and alpha bands, an anticorrelation in the same areas for delta band was found. Significant patterns of hemispheric asymmetry index have been reported, involving EEG bands that underlie cognitive impairments in ADHD. Alpha and theta bands were altered in the inattention group of patients, reflecting widespread deficiency of basic attentional processing.

## 1. Introduction

Attention-deficit/hyperactivity disorder (ADHD) is one of the most frequent childhood disorders, characterized by symptoms as hyperactivity/impulsivity and inattention that affect social and school/work functioning. The prevalence among adults is 2.5-4.9% [1]. The clinical phenotype moves along the domains of inattention or hyperactivity/impulsivity or a combination of both. Clinical research showed that the predominant features of adult ADHD are inattentive symptoms instead of hyperactivity/impulsivity symptoms, prevalent in childhood ADHD. This disorder has a pervasive symptomatologic expression associated with many brain structural and functional correlates. Neuroimaging evidence reported that physiopathology of ADHD exhibits abnormalities in brain areas mainly linked with cognitive control, attention, and motor functions [2–4]. Structurally, volume reduction in basal ganglia and subcortical structures as the putamen, caudate, globus pallidus [5, 6], nucleus accumbens, amygdala, and hippocampus [7] has been found. The putamen has been recognized as a primary structure in ADHD, since it has connections with cortical motor areas and is involved in higher-order cognitive functions like working memory [8]. Reduced volume has been considered to subtend asymmetry alterations. Shaw et al. [9] found increasing asymmetry in posterior temporooccipital areas due to reduced volume of prefrontal regions, and Dang et al. [10] found structural asymmetry in the caudate nucleus. Douglas et al. [11] reported increased volumetric asymmetry indexes in ADHD, also confirmed by white matter results, particularly in frontoparietal circuitry.

Brain connectivity studies hypothesized that dysfunction or disconnection between areas afferent to the default-mode network (DMN) might be one of the causes of ADHD, since the DMN involvement in cognition, attention, and motor functions [12]. Castellanos et al. [13] reported evidence for reduced functional connectivity (FC) between the anterior cingulate and two nodes of the DMN, the precuneus and the posterior cingulate cortex (PCC), and altered FC within the DMN regions (between the ventromedial prefrontal cortex and the PCC) also confirmed by Uddin et al. [14]. Other authors have suggested that the DMN is not altered at rest but in the transition from rest to task [15]. Abnormalities in the DMN are also evident in structural-functional right-based DMN asymmetry [10, 16]. Increased or atypical functional asymmetry and abnormal interhemispheric processing in ADHD have also been reported by Fassbender and Schweitzer [17].

Brain activity changes in patients with ADHD have been explored, also searching for different neuronal correlates trough two types of electroencephalography (EEG) analysis, clinical and quantitative. EEG's clinical approach consists of qualitatively observing EEG track, whereas quantitative EEG (qEEG) evaluates relative and absolute brain power across different frequencies band (delta, theta, alpha, beta, and gamma). The utility of EEG in ADHD diagnosis has been largely demonstrated [18–21] and recognized by the U.S. Food and Drug Administration (FDA, 2013). The EEG track analysis in children and adults with ADHD shows several differences compared to healthy controls [22]. Chabot and Serfontein [18], for the first time, demonstrated qEEG suitability in characterizing different neurophysiological ADHD profiles, later confirmed also by Clarke et al. [23, 24]. The authors identified three different EEG subtypes: the first characterized by an increased frontocentral theta/alpha ratio and a normal alpha power; the second by an increased theta/alpha ratio across all the scalp and decreased alpha mean power; the third subtype (combined) showed increased of frontal beta activity and decreased alpha power. Different models are proposed to characterize the EEG changes in ADHD patients. In particular, “Maturational States” that underline the increasing of theta activity as a consequence of a delay in cortical maturing in ADHD patients compared to healthy subjects [25]. Another model is “Developmental Deviation” model that proposed the increasing of theta activity in ADHD as a “Deviation” of normal brain maturation, and it is not comparable to the standard profile [18]. John et al. [26] demonstrated that children display a linear increase of theta and delta activity and, evolving through life, add an increase of alpha and beta bands. Alterations in brain asymmetry, mainly frontal alpha asymmetry, have also been reported by the EEG in ADHD patients. Frontal alpha asymmetry is the difference in the alpha band activity over right vs. left frontal hemispheres of the brain and is inversely related to cortical activation. Increased rightward alpha asymmetry in ADHD children during an eyes-closed resting condition was found [18]. These results were extended to adults by Hale et al. [27] that demonstrated a greater right frontotemporal lateralization of alpha activity during an eyes-closed condition. Adults with ADHD also displayed higher frontal alpha power and greater frontal alpha asymmetry associated with more severe ADHD symptoms [28].

As evidenced, ADHD is a heterogeneous disorder with different neurobiological and neurophysiological patterns [29]. With the aim at ameliorating the understanding of brain processing underpinning ADHD pathophysiology, the present study pointed to investigate the relationship between MRI and EEG resting-state functional asymmetry in different clinical profiles of ADHD.

## 2. Materials and Methods

**Figure 1:**
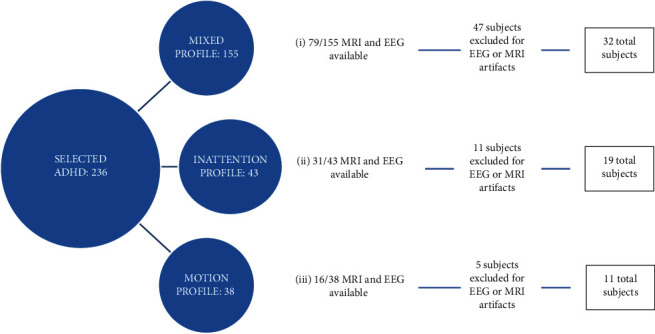
The procedure used for selecting the final samples of subjects.

The Healthy Brain Network Biobank of the Child Mind Institute is a dataset of subjects with different psychopathologies who underwent comprehensive psychiatric and neuroimaging assessments [30]. In the present study, subjects with ADHD symptoms were considered, based on the quotient ADHD system results. The procedure used for selecting the final samples of subjects is resumed in Figure 1.

## 3. The Quotient ADHD System

The quotient ADHD system is a clinical instrument that provides information about capabilities in controlling motor activity, attends to a task, and responds without impulsivity. It measures the adequacy of neurological control processes usually affected in ADHD. Scaled scores are reported as numerical values from 0-10, with the mean for individuals with ADHD are 6.8-7.1. Higher scaled scores indicate that the patient has a significant deficit in control of motion, attention, or both, associated with the scores more typically seen in individuals who will go on to meet diagnostic criteria for ADHD.

All participants were scanned on 3.0 Tesla scanners using standard resting-connectivity T2∗-weighted echo-planar imaging with anatomical T1 images (see Table 1 for further details).

All imaging data used is publicly available at the Neuroimaging Informatics Tools and Resources Clearinghouse (NITRC) (see http://fcon_1000.projects.nitrc.org/indi/adhd200).

## 4. Anatomical Data Preprocessing

The T1-weighted (T1w) image was corrected for intensity nonuniformity (INU) with N4BiasFieldCorrection [31], distributed with ANTs 2.2.0 [32] (RRID:SCR_004757), and used as T1w-reference throughout the workflow. The T1w-reference was then skull-stripped with a Nipype implementation of the antsBrainExtraction.sh workflow (from ANTs), using OASIS30ANTs as target template. Brain tissue segmentation of cerebrospinal fluid (CSF), white matter (WM), and gray matter (GM) was performed on the brain-extracted T1w using fast (FSL 5.0.9, RRID:SCR 002823) [33]. Brain surfaces were reconstructed using recon-all (FreeSurfer 6.0.1, RRID:SCR_001847) [34], and the brain mask estimated previously was refined with a custom variation of the method to reconcile ANTs-derived and FreeSurfer-derived segmentations of the cortical gray matter of Mindboggle (RRID:SCR_002438) [35]. Volume-based spatial normalization to one standard space (MNI152NLin2009cAsym) was performed through nonlinear registration with antsRegistration (ANTs 2.2.0), using brain-extracted versions of both T1w reference and the T1w template. The following template was selected for spatial normalization: ICBM 152 Nonlinear Asymmetrical template version 2009c [36] (RRID:SCR_008796; TemplateFlow ID: MNI152NLin2009cAsym).

## 5. Functional Data Preprocessing

Results included in this manuscript come from preprocessing performed using fMRIPrep 1.4.0 [37] (Esteban et al. [38]; RRID:SCR_016216), which is based on Nipype 1.2.0 [39, 40] (RRID:SCR_002502).

For each of the resting-state fMRI runs, the following preprocessing was performed. First, a reference volume and its skull-stripped version were generated using a custom methodology of fMRIPrep. The blood-oxygen-level-dependent (BOLD) reference was then coregistered to the T1w reference using bbregister (FreeSurfer) which implements boundary-based registration [41]. Coregistration was configured with nine degrees of freedom to account for distortions remaining in the BOLD reference. Head-motion parameters with respect to the BOLD reference (transformation matrices and six corresponding rotation and translation parameters) are estimated before any spatiotemporal filtering using mcflirt (FSL 5.0.9) [42]. BOLD runs were slice-time corrected using 3dTshift from AFNI 20160207 [43] (RRID:SCR_005927). The BOLD time-series (including slice-timing correction) were resampled onto their original, native space by applying a single, composite transform to correct head-motion and susceptibility distortions. The BOLD time-series were resampled into standard space, generating a preprocessed BOLD run in “MNI152NLin2009cAsym” space. Several confounding time-series were calculated based on the preprocessed BOLD: framewise displacement (FD), DVARS, and three region-wise global signals. FD and DVARS are calculated for each functional run, both using their implementations in Nipype [44] (following the definitions by Power et al. 2014). The three global signals are extracted within the CSF, the WM, and the whole-brain masks. Additionally, a set of physiological regressors were extracted to allow for component-based noise correction (CompCor) [45]. Principal components are estimated after high-pass filtering the preprocessed BOLD time-series (using a discrete cosine filter with 128 s cut-off) for the two CompCor variants: temporal (tCompCor) and anatomical (aCompCor). tCompCor components are then calculated from the top 5% variable voxels within a mask covering the subcortical regions. This subcortical mask is obtained by heavily eroding the brain mask, which ensures it does not include cortical GM regions. For aCompCor, components are calculated within the intersection of the aforementioned mask and the union of CSF and WM masks calculated in T1w space, after their projection to the native space of each functional run (using the inverse BOLD-to-T1w transformation). Components are also calculated separately within the WM and CSF masks. For each CompCor decomposition, the *k* components with the largest singular values are retained, such that the retained components' time-series are sufficient to explain 50 percent of variance across the nuisance mask (CSF, WM, combined, or temporal). The remaining components are dropped from consideration. The head-motion estimates calculated in the correction step were also placed within the corresponding confounds file. The confound time-series derived from head-motion estimates and global signals were expanded with the inclusion of temporal derivatives and quadratic terms for each [46]. Frames that exceeded a threshold of 0.5 mm FD or 1.5 standardized DVARS were considered as motion outliers. All resamplings can be performed with a single interpolation step by composing all the pertinent transformations (i.e., head-motion transform matrices, susceptibility distortion correction when available, and coregistrations to anatomical and output spaces). Gridded (volumetric) resamplings were performed using antsApplyTransforms (ANTs), configured with Lanczos interpolation to minimize the smoothing effects of other kernels [47]. Nongridded (surface) resamplings were performed using mri_vol2surf (FreeSurfer).

As final step, a high-pass filter was applied to fMRI time courses to linearly detrend the signal.

## 6. EEG Data Processing

All participants underwent a five-minute resting-state recording session. In particular, participants viewed a fixation cross on a blank background and were instructed to try not to think of anything for the duration of the scan. EEG signals were recorded using a 128-channel EEG Geodesic HydroCel System by EGI. Recording reference is at Cz. The impedance of each electrode is kept below 40 kOhm. Impedance is tested during the recording section. Ocular artifacts were removed by linearly regressing the EOG channels from the scalp EEG channels [30]. EEG signals were preprocessed with the MATLAB toolbox Fieldtrip (Donders Centre for Cognitive Neuroimaging, Nijmegen, Netherlands). EEG was resampled at 500 Hz, filtered and linearly detrended, zero-padded, and smoothed using a 1 Hz taper.

Spectral decomposition was performed using the multitaper method implemented in the ft_freqanalysis function of the FieldTrip analysis package [48]. For each participant, according to standard frequency bands (delta (1–3 Hz), theta (4–8 Hz), alpha (8–14 Hz), and beta (13–30 Hz)), power spectral analysis was performed in all EEG channels (power-EEG). Fifteen channels were selected from each hemisphere (15 left and 15 right); specifically, we selected three channels for different scalp areas (frontal, central, temporal, parietal, and occipital).

## 7. Definition of Brain Regions

Analysis of EEG power activity was performed into distinct regions of interest (ROI), following the international 10-20 system and ensuring associated brain region obtained with structural MRI. From the original 128 EEG channels, 15 channels were selected and then grouped into five areas for each hemisphere (frontal: F3, F4, F6, F7, F8, and F9; central: C1, C2, C3, C4, C5, and C6; temporal: T5, T6, T8, T9, T10, and T11; parietal: P1, P2, P3, PP8, P9, and P10; and occipital: O1, O2, PO3, PO4, PO7, and PO8), for a total of 3 channels per area. Preprocessed time (time-EEG) and power series (power-EEG) have been carried out for each channel and averaged for each area; for the metric of power-EEG, different bands were calculated: beta, gamma, theta, delta, and alpha.

Regarding MRI, 84 cortical and subcortical parcels (42 from left hemisphere and 42 from right hemisphere), derived from structural MRI processing, have been considered for the analysis; in order to match and overlap on EEG areas, 33 parcels for each hemisphere were chosen and then grouped into five areas (frontal: caudal anterior cingulate, caudal middle frontal, lateral orbitofrontal, medial orbitofrontal, pars opercularis, pars orbitalis, pars triangularis, rostral anterior cingulate, rostral middle frontal, superior frontal, and frontal pole; central: paracentral, postcentral, and precentral; temporal: bankssts entorhinal fusiform inferior temporal, middle temporal, parahippocampal, superior temporal, temporal pole, and transverse temporal; parietal: inferior parietal, isthmus cingulate, posterior cingulate, precuneus, superior parietal, and supramarginal; and occipital: cuneus, lateral occipital, lingual, and pericalcarine). Resting-state fMRI data have been preprocessed and averaged across the ten areas by mapping each parcel to the corresponding lobe, excluding subcortical parcels.

## 8. Asymmetry Analysis

The functional connectome of MRI (FC-MRI) consists of a 42 × 42 connectivity matrix, where the strength of connectivity between each pair of network nodes has been defined as the Pearson correlation of the resting-state fMRI time course averaged across the 42 anatomical parcels.

On the other hand, the functional connectome of EEG (FC-EEG) consists of a 15 × 15 connectivity matrix, where the strength of connectivity between each pair of network nodes has been defined as the Pearson correlation of the time-EEG time course averaged across the 15 channels.

For each brain node, the hemispherical degree of centrality has been estimated as the sum of the strength of connectivity between the node and all the contralateral nodes.

The asymmetry index (AI) is designed to find the differences between the right and left hemispheric functional networks. The AI of the brain networks was calculated according to Argyropoulos et al. [49] with the following formulation: AI(*X*) = 100 × (∣*X*(R)∣−∣*X*(L)∣)/*N*, where *X*(R) and *X*(L) represent the degree of centrality absolute value of the right and left hemispheres, respectively. *N* represents the number of parcels/channels for each hemisphere, 42 for MR-FC and 15 for EEG-FC. The range of AI(*X*) is from +100 to −100. When AI(*X*) is positive, this indicates that the brain network showed prominent rightward asymmetry; when AI(*X*) is negative, it indicates that the brain network showed leftward asymmetry.

## 9. Statistical Analysis

Evaluation of the asymmetry of three different metrics (FC-fMRI, FC-EEG, and power-EEG), for every group of subjects, was obtained in order to test how the asymmetry score covariates across both modalities and metrics. To test whether different groups of ADHD patients can be discriminated against by AI, the data were first tested for normality with the Shapiro-Wilk test. Then, ANOVA has been performed to evaluate the difference between EEG and fMRI indices measures within the three subgroups, corrected for Tukey's multiple comparisons. Then, in order to consider the shared variance across brain regions, partial least squares (PLS) with principal component regression is an analytical method useful for understanding the relationships between multiple correlated variables, and, for each group of subjects, correlations have been calculated to evaluate the relationship between EEG and fMRI asymmetry scores across subjects belonging to “inattention,” “motion,” and “mixed profile” groups, separately.

In the absence of a control group, real networks were compared to random networks, with the aim of validating whether the asymmetry characteristics are at least different then chance. To this purpose, we randomized the network using Maslov's wiring algorithm implemented in GRETNA toolbox [50] and then compared the distributions of the network metric in randomized and real graphs.

## 10. Results

The final samples of subjects with suitable data were as follows: inattention profile 19 subjects (7 females; mean age: 34.7 ± 10.7; mean quotient score: 8.2 ± 1.1); motion profile 11 subjects (5 females; mean age: 31.4 ± 10.9; mean quotient score: 8.7 ± 1); and mixed profile 32 subjects (8 females; mean age: 25.3 ± 7.6; mean quotient inattention score: 9.1 ± 1).

AI was calculated for each of the three metrics (FC-fMRI, FC-EEG, and power-EEG) for every subject belonging to “inattention,” “motion,” “mixed profile,” and ADHD total group (which is the sum of every subject belonging to subgroups). For the “inattention” group of patients, AI from FC-fMRI has a leftward representation in the frontal area, and rightward representation in central, temporal, parietal, and occipital areas. AI from power-EEG has a whole-brain leftward representation for theta band and whole-brain rightward representation for delta band, whereas alpha band had leftward prevalence in frontal, central, and temporal areas and rightward prevalence in parietal and occipital areas.

**Figure 2:**
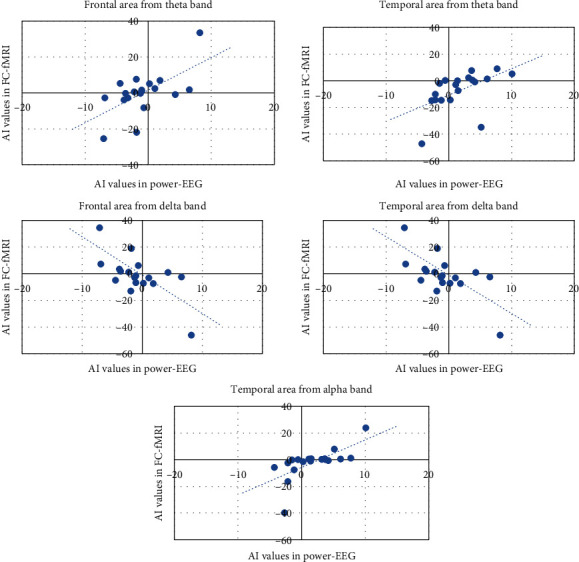
Scatter plots of asymmetry index (AI) of FC-fMRI versus power-EEG in brain areas and EEG bands exhibiting significant correlation in the inattention group.

AI significantly correlated across subjects between fMRI and power-EEG in the inattention group in frontal area (*r* = 0.61, *p* = 0.005) with a goodness of fit from PLS of *R*^2^ = 0.63 (*p* = 0.013), and temporal area (*r* = 0.53, *p* = 0.019) with a goodness of fit from PLS of *R*^2^ = 0.57 (*p* = 0.034) for brain theta band; a negative correlation was found in frontal (*r* = −0.61, *p* = 0.005) with a goodness of fit from PLS of *R*^2^ = 0.51 (*p* = 0.069) and temporal areas (*r* = −0.80, *p* = 0.00003) with a goodness of fit from PLS of *R*^2^ = 0.71 (*p* = 0.003) for brain delta band; finally, a positive correlation in temporal area (*r* = 0.67, *p* = 0.002) with a goodness of fit from PLS of *R*^2^ = 0.66 (*p* = 0.009) for brain alpha band was observed (Figure 2).

**Figure 3:**
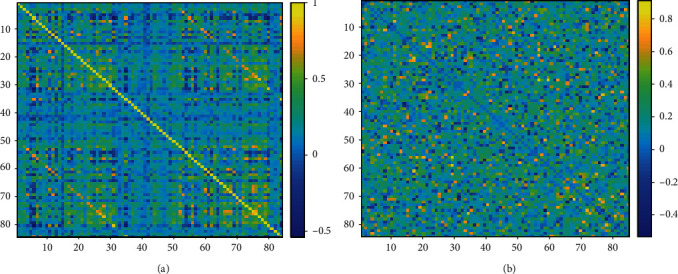
Functional connectivity matrix of real network (a) and randomized network (b) calculated in a random patient. The colorbar indicates the strength of connectivity between brain parcels.

Regarding FC-EEG, FC-fMRI, and power-EEG metrics, no statistical difference from ANOVA was found between groups. Moreover, the correlations from the randomized network showed no statistical significance using the AI generated from randomized network between the three metrics (FC-fMRI, FC-EEG, and power-EEG) (Figure 3).

## 11. Discussion

The present study aimed at investigating, for the first time, the correlation between resting-state functional asymmetry indices derived from fMRI and EEG in ADHD patients. The clinical sample has been categorized in the three profiles: motion, inattention, and combined, according to the prevalent symptomatology. Significant results were selectively observed in the inattention group of patients where we reported two patterns of correlations between these two measures, within the same regions. Positive correlations between fMRI and EEG asymmetry indices were found in frontal and temporal areas for theta band, whereas a negative correlation was found in the same areas for delta band. In the temporal area, a positive correlation between EEG and fMRI asymmetry indices occurred for alpha band. Theta band reflects slow brain activity (ranges from 4 to 7.5 Hz), whereas the alpha reflects more rapid brain activity (ranges from 7.5 to 12.5 Hz); usually, the power of these bands responds in opposite ways, namely when one band increases, the other one decreases [51]. In our sample, this usual anticorrelation disappears, since for both bands, a positive correlation between the two measures of brain activity was found. This result could reflect an alteration of normal relationships between brain activity in ADHD pathology. It is noteworthy and interesting that these bands underpin to processing of attentional stimuli. Alpha band oscillations seem to respond to a great variety of cognitive domains and are closely associated with attention, specifically on focusing and blocking information processing. The alpha band is not related to attention in general; in fact, the monitoring of new information is majorly associated with the theta, which entails an increase of this band activity [52]. These data suggest that ADHD patients with the inattention predominant profile report a disorder of the normal relationships between the bands that are more active during attentional processing. One interpretative hypothesis may attribute this alteration to a great deal of concentration required to the patients in processing of attentional stimuli. Furthermore, in the inattention group, AI from power-EEG revealed a whole-brain left representation for theta band and whole-brain right representation for delta band, whereas alpha band had leftward prevalence in frontal, central, and temporal areas and right prevalence in parietal and occipital areas. Similarly, Hashemi et al. [53] in a large sample of healthy subjects found that asymmetry in frontal areas was more pronounced on the left side (reflecting greater neural activity in the right hemisphere) whereas in temporoparietal areas showed the opposite pattern.

In children with ADHD, interhemispheric EEG asymmetry for all bands was found in frontal, temporal, and occipital regions that suggested a decrease in coordination of neurophysiological activity between hemispheres [18]. Lazzaro et al. [54] performed EEG bands power analysis in 26 male adolescents with ADHD showing an increased absolute theta and alpha activity that suggested a possible continuation of maturational lag in frontal regions.

Bresnahan et al. [55] for the first time investigated EEG profiles also in adults with ADHD, comparing them with children and adolescents and with controls. The results demonstrated that theta power activity remained elevated in all subjects' groups, but there was a decrease in relative beta activity with age. These results also indicate that variations in the qEEGs subjects in relative beta power are age-related and reflect some of the age-related symptoms reported in the clinical setting linked to hyperactivity. As presented in the introduction, in people with normal development, it is not observed theta increase, which is typical of pathological profiles as Alzheimer disease or specific of elderly people.

Our results, observed in groups of adults with ADHD, sustain the hypothesis about a maintenance of bands activity variations across the growing. In particular, alpha and theta band alterations seem to be a stable tract in ADHD pathology, specifically for group with prevalence of inattention deficit.

At a descriptive level, it is useful to report that ADHD patients display brain structural alterations that potentially generate abnormal left-right EEG coherence [56]. Barry et al. [57] found differences in interhemispheric coherence for theta band in children with ADHD. These results could be attributable to reduced development and specialization of hemispheres in ADHD, with consequent alteration of neuronal circuits involving theta activity. In particular, theta activity seems to be generated in the hippocampus during learning of episodic memory, and it is implicated in hippocampal-neocortical circuits for memory consolidation [58].

The present data strengthens Hale et al.'s [59] proposal that frontal alpha asymmetry is an endophenotypic characteristic in ADHD and also supports the hypothesis about a diffuse alteration of EEG measures of brain activity, recruiting also other bands. Frontal asymmetry has been previously reported in ADHD [28, 60], but only a few studies examined frontal alpha asymmetry in ADHD. Alperin et al. [61] in a large sample of young focused on the role of EEG to investigate patterns of atypical right asymmetry as a possible endophenotype of ADHD. Within the available studies, some reported right alpha symmetry across samples of patients with different ages [27, 62], whereas others reported left alpha asymmetry [55, 63]. Moreover, alpha power seems to be closely related also with emotional aspects. It has been reported that right asymmetry is mainly consistent with dysregulation of positive affect, while negative affect is related to left asymmetry [64].

The inverse correlation observed in healthy subjects between delta band, which also reflects slow brain activity (ranges from 0.5 to 4), and alpha band, is maintained in our sample, probably because delta band is less specifically associated with ADHD pathology for alpha and theta bands. Delta band is less studied for alpha and theta, because it is more subject to artifacts by ocular movements. In normal subjects, delta band is considered a marker for cerebral immaturity, is related to brain lesions, and is considered a measure of disruption of normal brain functioning.

In conclusion, this is the first study investigating specific brain activity alterations related to several types of ADHD patients, both with EEG and fMRI. Significant patterns of hemispheric asymmetry have been reported, involving EEG bands that specifically underlie cognitive impairments in ADHD. Alpha and theta bands, selectively associated with this cluster of disorder, were altered in inattention group of patients, reflecting widespread deficiency of basic attentional processing that is maintained during life. In addition, delta band alteration, even if nonspecific with respect to ADHD, corroborates the diffuse impairment of cortical brain activity as an endophenotypic tract of ADHD.

## Figures and Tables

**Table 1:** MR acquisition parameters of the “Staten Island” site.

	Slices	% FOV phase	Resolution (mm)	TR (ms)	TE (ms)	TI (ms)	Flip angle (°)	MultiBand Accel.
T1 MPRAGE	176	100%	1.0 × 1.0 × 1.0	2730	1.64	1000	7	N/A
fMRI	54	100%	2.5 × 2.5 × 2.5	1450	40.0	N/A	55	3

## Data Availability

The data used to support the findings of this study have been deposited in the http://fcon_1000.projects.nitrc.org/repository (Alexander, L. et al. An open resource for transdiagnostic research in pediatric mental health and learning disorders. Scientific Data 4, 170181 (2017)).
